# Phage Products for Fighting Antimicrobial Resistance

**DOI:** 10.3390/microorganisms10071324

**Published:** 2022-06-30

**Authors:** Yuanling Huang, Wenhui Wang, Zhihao Zhang, Yufeng Gu, Anxiong Huang, Junhao Wang, Haihong Hao

**Affiliations:** 1 National Reference Laboratory of Veterinary Drug Residues, Huazhong Agricultural University, Wuhan 430070, China; huangyuanling0226@163.com (Y.H.); wwh6572@163.com (W.W.); zhangzhihao@webmail.hzau.edu.cn (Z.Z.); guyufeng@webmail.hzau.edu.cn (Y.G.); anxionghuang@webmail.hzau.edu.cn (A.H.); wangjunhao@webmail.hzau.edu.cn (J.W.); 2 MOA Laboratory for Risk Assessment of Quality and Safety of Livestock and Poultry Products, Huazhong Agricultural University, Wuhan 430070, China; 3 Shenzhen Institute of Nutrition and Health, Huazhong Agricultural University, Shenzhen 518000, China; 4 Shenzhen Branch, Guangdong Laboratory for Lingnan Modern Agriculture, Genome Analysis Laboratory of the Ministry of Agriculture, Agricultural Genomics Institute at Shenzhen, Chinese Academy of Agricultural Sciences, Shenzhen 518000, China

**Keywords:** antimicrobial resistance, phage products, advantages and disadvantages of phage therapy, development prospects

## Abstract

Antimicrobial resistance (AMR) has become a global public health issue and antibiotic agents have lagged behind the rise in bacterial resistance. We are searching for a new method to combat AMR and phages are viruses that can effectively fight bacterial infections, which have renewed interest as antibiotic alternatives with their specificity. Large phage products have been produced in recent years to fight AMR. Using the “one health” approach, this review summarizes the phage products used in plant, food, animal, and human health. In addition, the advantages and disadvantages and future perspectives for the development of phage therapy as an antibiotic alternative to combat AMR are also discussed in this review.

## 1. Introduction

Antimicrobial resistance is a naturally evolving phenomenon that emerged soon after the discovery of penicillin in 1940 [1]. Antibiotics are highly efficient against bacterial infections, saving millions of lives and drastically reducing mortality rates. However, multidrug-resistant bacteria (MDR), extensively drug-resistant bacteria (XDR), and even pan-resistant bacteria (PDR) have evolved as a result of antibiotic overuse, abuse, and misuse. In particular, ESKAPE bacteria seriously threaten human health worldwide. According to the latest estimates, approximately 700,000 people worldwide die directly from AMR bacteria each year, with that number possibly rising to 1 billion by 2050 [2]. AMR is one of the top ten global public health threats facing humans, according to the WHO. As a result, FAO, WOAH, and WHO attach great importance to this and jointly launched the “one health” approach to combat AMR [3]. The interdependent relationship between the food chain and the environment makes resistant bacteria widespread in plants, animals, food, and humans, and the “one health” approach trinity model is ideally suited to address AMR [4,5]. Phages are currently one of the antibiotic alternatives with the most potential because of their ability to effectively combat bacterial infections. Phages are a new alternative therapy under the “one health” approach that can be used to control bacteria in plants, animals, food, and humans [6]. Currently, phage therapy is emerging globally, and in this review, we summarize the application of phage products for plants, animals, food, and human health from the perspective of “one health” from the two databases of phage companies and bacteriophage news [7,8]. Furthermore, the advantages and disadvantages of phages as antibiotic alternatives to combat AMR and their future development prospects are also discussed in detail.

## 2. Phage Biology

As early as 1896, Ernest Hankin discovered antibacterial substances against *Vibrio cholerae* from water extracted from the Ganges and Jumna rivers in India, laying the foundation for the subsequent discovery of phages [9,10]. The term “phage” was introduced by Félix d’Hérelle after he discovered the “anti-microbe” *Shigella* in 1917 [11]. Phages are abundant entities on the planet, with a population of 10^31^, which is 10–100 times that of their obligatory parasitic host bacterium [12]. The genome of phages is composed of single-stranded (ss) or double-stranded (ds) DNA or RNA, which is encapsulated by a wide variety of protein capsids. The universal viral taxonomy established by the International Committee on Taxonomy of Viruses (ICTV) divides phages into polyhedral, filamentous, pleomorphic, and tailed according to capsid morphology [13]. Phages can be classified into temperate and virulent based on their life cycle and reproductive characteristics. However, the process of bacterial infection is different between temperate and virulent phages.

**Figure 1:**
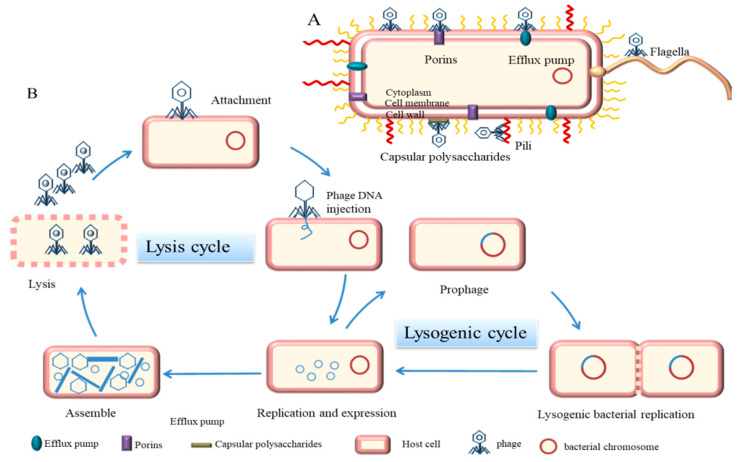
Mechanism of phage infestation of bacteria. (**A**) Receptors for phage adsorption on bacteria (purple represents porins, blue represents efflux pumps, yellow represents flagella, red represents pili, and gray represents capsular polysaccharide) (**B**) Described five phases of a phage lytic cycle and a lysogenic cycle.

Virulent phages enter the lytic cycle, which usually consists of five stages. The tail filament first adsorbs to a specific receptor on the surface of the host bacteria. These receptors can be located on cell walls, capsular polysaccharides, outer membrane proteins, efflux pumps, or appendages, such as pili and flagella [11] (Figure 1A). Second, the phage-derived enzymes (such as endolysins) lyse the peptidoglycan of the cell wall and the tail pipe penetrates through the cell membrane to inject its DNA into the host bacteria [14]. Third, phages perform biosynthesis, such as nucleic acid replication, RNA transcription, and protein translation in dormitory cells. Fourth, phages assemble into progeny phages. Finally, when the number of progeny phages reaches a certain threshold, bacteria lyse and release progeny phages [15] (Figure 1B). For Gram-negative bacteria, lysis is achieved by three different functional proteins, holins, endolysins, and spanins, which act on the inner membrane, peptidoglycan, and outer membranes of the cell envelope, respectively [16]. Temperate phages differ from virulent phages in a number of important ways. For example, temperate phages integrate their genomes into the chromosomes of host bacteria, which do not lyse but enter the lysogenic cycle. Phages grow and multiply with host bacteria [17]. Under certain conditions, temperate phages can also enter the lytic cycle, depending mainly on phage-encoded repressors and regulators, as well as the control of phage enzymes [18]. For example, under stress responses and light, temperate phages can initiate the expression of lytic genes. Temperate phages can regulate the gene expression and behavior of bacteria through different mechanisms and enhance phage-host fitness [19]. In addition, both virulent and temperate phages have a pseudo-lysogenic nature, which means that viral DNA is present in the host bacteria in a form similar to a plasmid and the host at this moment is only the vector of the phage [14,18].

The key to killing bacteria by phages depends on lysing the bacterial cell wall and virulent phages are generally selected for treatment. It has commonly been assumed that icosahedral DS DNA phages containing tails can effectively treat human and animal infections [20]. Bacteria may acquire resistance genes or genes with pathogenic potential after the lysogenic transformation of temperate phages and they are generally not recommended for therapeutic purposes [11]. However, with advances in synthetic biology, temperate phages can be designed to interfere with bacterial intracellular processes and cause bacterial cell death. Alternatively, genomes of temperate phages were engineered to eliminate known virulence genes involved in the lysogenic cycle [21]. The current crisis of AMR makes phage therapy re-emerge globally and the cases of phage therapy in preclinical research are also gradually increasing [22,23,24].

## 3. Phages Products in Plant Health

More than 200 plant bacteria have been reported to cause significant crop losses during preharvest, storage, and transport [25]. Antibiotics have also been used against plant pathogens since World War II, and AMR has been widespread in some plants and crops due to the dissemination of resistance genes in the environment. For example, antibiotic resistance genes (strAB) have emerged in *Pseudomonas syringae*, *Xanthomonas campestris*, and *Erwinia amylovora*, triggering resistance to streptomycin [26]. The first experimental evidence that phages may be associated with plant pathogenic bacteria was the discovery that filtrates obtained from cabbage were able to inhibit cabbage decay caused by *Xanthomonas campestris* pv. [27]. Subsequently, in 1925 Kotila and Coons used phages to prevent soft rot caused by *Pectobacterium atrosepticum* and *Pectobacterium carotovorum subsp* on potato tuber and carrot slices, respectively. In 1935, Thomas used phage against the phytopathogen *Pantoea stewartii* to significantly reduce the incidence [28]. Modern studies have shown the effectiveness of phages for plant health and can target drug-resistant plant-bacteria with extremely high efficiency. For example, the isolation of a novel phage Xoo-sp2 infected with *Xanthomonas oryzae* from soil can effectively control bacterial blight in rice [29]. The engineered phage Y2 can effectively control and rapidly detect *Erwinia amylovora*, a fire blight pathogen [30]. Recently, three phage cocktails (φEa2345-6, φEa1337-26, and Eh21-5) are effective against fire blight in apples and pears. Four phage cocktails (Eram2, Eram26, Eram24, and Eram45) are effective against fire in blighted pears [31]. *Xylella fastidiosa (Xf)* is a novel plant pathogen with a wide range of plant hosts and a spectrum of insect species that are now causing significant damage to world agriculture. Phage is a novel therapy to control diseases caused by *Xf* [32,33]. Given the current invasion of *Xf* into agroecosystems, these phages can be implemented as biological agents and are excellent candidates for development into phage cocktails.

As phage studies advance, the number of phage products targeting plant pathogens in the market is also increasing. At present, the United States Environmental Protection Agency (USEPA) has approved several phage products to fight plant pathogens, and commercial phage products are summarized in Table 1. Only six phage products for plant health have been commercialized, mostly for *soft rot Enterobacteriacea*, *Clavibacter michiganensis*, *Xanthomonas citri*, and *Erwinia amylovora*, which are all prevalent plant pathogens. OmniLytics (Kuala Lumpur, MY, USA) was the earliest registered phage-based biopesticide product and its AgriPhage™ product line has been approved by the USEPA for the control of bacterial diseases in citrus, tomato, apple, and pear. In addition, the product also obtains an Organic Materials Review Institute (OMRI) listing for commercial organic growers. Enviroinvest Erwiphage PLUS (Hungary) is the second company to obtain a biopesticide registration and its product Erwiphage can fight fire blight caused by Rosaceae plants. The Biolyse^®^ BP product, developed by APS Biocontrol (Dundee, UK), is a phage-based potato tuber wash for the prevention of soft rot during storage. Fixed-Phage (Glasgow, UK) also has a product for a variety of bacteria (agriPHIX™) that plays a significant role in improving the storage of a range of crops. In conclusion, there are a few phage products for plants that need to be further developed.

## 4. Phage Products in Animal Health

The indiscriminate and extensive use of antibiotics in animals has been one of the principal reasons for the rapid spread of AMR. The new EU law prohibiting the prophylactic use of antibiotics in farmed animals was implemented in 2022 and the use of antibiotics is also strictly regulated in the United States and Canada [39]. The recent resurgence of phage therapy has also prompted the extensive application of phages in veterinary medicine. The first known therapeutic use of phages in veterinary medicine was associated with Felix d’Herelle, who used phages in 1919 to prevent and treat *Salmonella* infections in chickens and effectively reduce mortality in chickens [40]. However, when Pyle used phage therapy in 1926 to treat *Salmonella* Enteritidis infection in chickens, the results were less than encouraging [41]. Until the 1980s, William Smith reconsidered the use of phage therapy in animals and experimented with chickens, cattle, and pigs [42]. The early British team conducted a small clinical trial of a phage cocktail for canine otitis media caused by *P. aeruginosa* in 2010 and the results were greatly encouraging [43]. Subsequently, the application of phages in animals has been increasing, mainly for treating *E. coli* and *Salmonella* infections in poultry and pigs, as well as mastitis in cattle caused by *S. aureus* [44]. In aquaculture, phages are also effective against *Vibrio*, *Pseudomonas*, and *Aeromonas*, reducing fish mortality [45].

**Figure 2:**
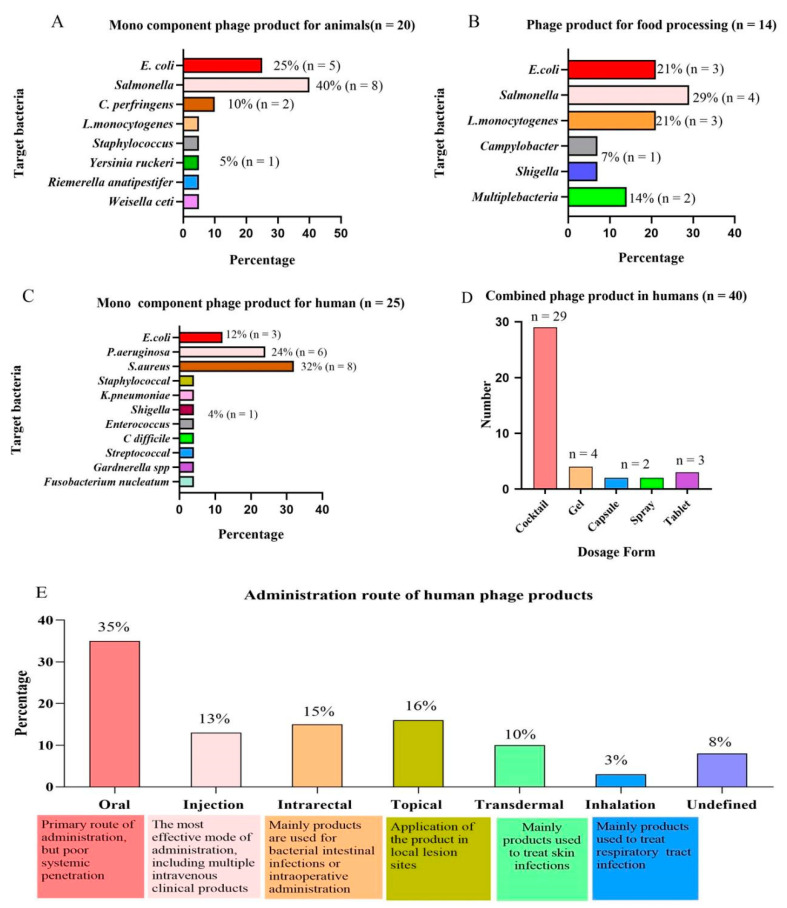
Analysis of phage products from the perspective of the “one health” approach. (**A**) Targeted bacteria for mono-component phage products in animals. (**B**) Targeted bacteria for phage products in food processing. (**C**) Targeted bacteria for mono-component phage products in humans. (**D**) Main dosage forms of phage products in human therapy. (**E**) Main routes of administration for human phage products.

The preharvest application of phages can effectively reduce the infection and colonization of live poultry and minimize the risk of pathogens entering the food chain, thereby reducing the infection of zoonotic bacteria [46]. At present, phage products in animals mainly focus on the preharvest application of livestock, poultry, and the clinical application of pets. The detailed commercial phage products used in animals are shown in Table 2. A total of 9 of the 38 veterinary phage products counted have been approved by the FDA and 3 by the European EFSA. The products developed by Intralytix (Baltimore, AR, USA) mainly focus on pet food safety and preharvest intervention. The product line of PhagePharm (Qingdao, China) focuses on common pathogens in the environment and is used as an environmental improver, and the product line of Fixed-Phage (UK) is mostly phage cocktails. Figure 2A reveals that 20 mono-component phage products in the market almost target *E. coli, Salmonella,* and *C.* *perfringens* in poultry. A variety of phage cocktail products also target the infection of these bacteria. Most veterinary phage products are mainly in the form of food additives in animal feed or drinking water to prevent and control bacterial diseases. Only a few phage products are made into gel formulations for topical epidermic medication, such as Staphage Lysate (SPL)^®^, a phage product from Delmont Laboratories (Swarthmore, PA, USA), which is also the only *staphylococcal* product approved for use in *Staphylococcus* canis skin infections [47]. In contrast, phage products targeting companion animals remain to be further investigated, especially the study of bacterial dermatology, which may have significance in the future.

## 5. Phages Products in Food Health

The use of antibiotics aggravates AMR in livestock and poultry products and the high morbidity and mortality caused by foodborne pathogens has been a global burden [68]. Contamination caused by foodborne pathogens can be transmitted from production lines to humans, ultimately threatening human health. Phages are desirable for the biological control of foodborne pathogens as an effective natural and ecological alternative [69]. There are also increasing studies on the effectiveness of phages against foodborne pathogens. For example, Mengzhe demonstrated that phage STP4-A with a wide host range is effective against *Salmonella* as a food additive [70]. Vikram demonstrated that phage preparation can effectively reduce the level and prevalence of *E. coli O157:H7* in food [71]. As early as 1958, the U.S. Food and Drug Administration (FDA) recognized phages and their derivatives as GRAS (generally recognized as safe) through the Food Additives Amendment to the Federal Food, Drug, and Cosmetic Act [72]. Phages are primarily used in three departments: primary production, biological preservation, and biological harmlessness in the food industry to ensure food safety [53]. Phages used in primary food production can prevent foodborne pathogens from entering the human body through the food chain, which is an excellent pre-harvest strategy. Livestock and poultry products are contaminated with pathogens in the production, processing, distribution, and consumption links. The application of phage products in postharvest can effectively reduce the presence of pathogens on carcasses, packaging, and RTE poultry products [68]. The benefit of phages for postharvest poultry processing is that they do not affect the quality senses and nutritional value of food [48].

Phage products are currently used with high safety to eliminate pathogens in animal food (meat products and dairy products) and plant food (fruits and vegetables). The FDA has granted phage products GRAS approval, allowing them to be used in livestock and poultry products. The use of phage products in food is also approved by health agencies in Israel, Canada, China, Switzerland, Australia, New Zealand, and the European Union [53]. Since the FDA approved the first phage product, ListShield™, as a food preservative in 2006, a significant number of phage products have emerged worldwide to combat foodborne pathogens [34,73]. As of November 2021, 14 phage products have been used in food processing, of which 11 have been approved by the FDA, including Intralytix (USA) and Micreos (Utrecht, Netherlands). Table 3 lists the commercial phage products used to combat foodborne pathogens in detail. The statistics of approved commercial phage products against foodborne pathogens revealed that Intralytix (USA) has made remarkable contributions to the field, with five products for marketing, and has gained Jewish cleansing and halal certification. Figure 2B reveals that commercial phage products primarily compete with *E. coli*, *Salmonella*, and *L*. *monocytogenes*, which seriously threaten human health. It is worth noting that *Campylobacter* is the most commonly reported foodborne pathogen, but there are no commercial *Campylobacter* phage products. A recent project (C-SNiper) directed by the Spanish Technology Center (AZTI) developed a prototype phage product for *Campylobacter* that is expected to be globally commercialized in 2022 [74].

## 6. Phage Products in Human Health

The application of phages to treat human diseases dates back to the successful injection of phage preparations in France in 1921 to treat five children with dysentery caused by the *Shigella* infection [24]. Belgian researchers published the first paper in the same year on the successful use of phages to treat furuncles and carbuncles of human skin [9]. Initially, the French company L’Oréal sold five phage preparations for the treatment of bacterial infections, Antipiol (Deutsch, Germany) produced Enterofagos, and EliLily (Indianapolis, IN, USA) first sold “Staphylofel” phage preparations for the treatment of *streptococci* and *E. coli* [86]. D’Herelle and Eliava first used phages to control cholera in India in 1931 and found no side effects following treatment [87]. During World War II, phages were also applied by both Soviet and German armies to treat wound infections, with the German army using *Shigella* phage preparation “Polyfagin” by Behringwerke Leverkusen to treat and prevent dysentery in soldiers [88]. In the late 1930s, however, the Committee on Pharmacy and Chemistry of the American Medical Association stated that the efficacy of phage therapy was unclear and further research was needed. Together with the discovery of penicillin, which led to the successful introduction and widespread use of antibiotics, interest in phage therapy has diminished, with only the Soviet Union and some countries in Eastern Europe still investigating it [14].

Common infections or minor injuries may be fatal with the increasing threat of AMR to humans. Researchers found great potential for phage therapy and phage therapies are increasingly being used for human bacterial diseases. In 2000, clinical human trials using phage therapy as a potential antibiotic alternative officially began in the United States, and phase I clinical data was first published in 2009. Clinical trials have revealed that phage cocktails against *E. coli, S. aureus, and P. aeruginosa* are safe for the treatment of wounds [89]. In 2013, the European Commission supported the large multinational phage therapy multicenter clinical research program “Phagoburn”, which treated 27 patients infected with *P. aeruginosa* burn wounds with phage therapy in France, Belgium, and Switzerland [90]. Despite the intended purpose not being achieved, this is the first time that three national regulatory agencies reached a consensus about phage cocktails for human therapy. At present, there are five phage therapy institutions worldwide, which are: Eliava Phage Therapy Center (Tbilisi, Georgia), Phage Therapy Center (Tbilisi, Georgia), Center for Innovative Phage Applications and Therapeutics (West Philadelphia, PA, USA), Phage International (San Ramon, CA, USA), and Phage Therapy Unit (Wrocław, Poland). Eliava Phage Therapy, founded in 1923, was the first institution to focus on phage therapy and has marketed phage cocktail products targeting specific pathogenic bacteria to treat human bacterial infections [91].

It has been confirmed that phage therapy has a lethal impact on a range of bacteria, which has contributed to an increase in phage therapy research and development for human diseases by multiple institutions around the world. However, no phage products have been approved for human use in the European Union or the United States. The FDA has merely opened up the regulatory pathway for phages to provide a green channel for phage products for clinical use in emergencies. Phage therapies are approved for use in emergency treatment plans in the European Union, Australia, France, and Belgium [92]. Detailed information on phage products currently approved and in preclinical studies worldwide is provided in Table 4. Figure 2C reveals that phages in preclinical products are almost exclusively targeted at MDR bacteria, especially “ESKPAEE” pathogens, including *E. faecium*, *S. aureus*, *K. pneumoniae*, *E. coli*, and others. It can treat the infections caused by these bacteria at different sites, including bone and joint infections (IOA), diabetic foot ulcers (UPD), and MARS caused by *S. aureus* [93,94]. It can treat the fibrosis and burn infections caused by *P. aeruginosa* [95]. It can treat urinary tract infections and IBD caused by *E.coli* and *K. pneumonia* [96,97]. Among the preclinical phage products, the cocktail products composed of 4–8 phage mixtures account for 60% of the total. 

Phage cocktails can increase the host range and avoid targeting a specific pathogen. In addition, rapid identification of bacterial pathogens is a time-consuming and laborious process before individualized treatment with phages [121]. Notably, phage cocktails are still targets for treating bacterial diseases caused by “ESKPAEE” pathogens. Figure 2D reveals that phage cocktail products that have been marketed in Russia and Georgia are also basically liquid phage cocktails, with only a few gels, capsules, and tablets available. Due to the effective identification of phages in the reticuloendothelial system, the half-life of phages in humans is usually relatively short [122]. Figure 2E reveals that the route of administration has a significant impact on the efficacy of phage absorption into the human body. Currently, the administration routes of phage products in preclinical studies mainly include oral, topical, transdermal, inhalation, and intrarectal administration. There are various routes of administration for phage products, with oral administration accounting for 35% of the total and remaining the most prevalent, followed by topical and intrarectal administration. Oral administration is effective in delivering phages to the gastrointestinal tract but it is the least effective route for systemic penetration. The most effective mode of delivery is an injection, which may deliver phages to practically all organs and tissues in minutes. Therefore, the efficacy of phage therapy is determined by the route of administration.

Endolysin and virosome-associated lysozyme (VAL), which are phage-derived peptidoglycan-degrading enzymes, are also bactericidal. Endolysins are enzymes used by phages to lyse the bacterial cell wall at the end of the replication cycle, while the VAL is responsible for the injection of genetic material into infected cells for peptidoglycan degradation [18,123]. Many studies on the antibacterial effect of endolysin are currently being implemented in human medicine clinics. Endolysin is also one of the alternatives to antibiotics. It has the advantages of killing the host quickly, host specificity, preservation of the normal microbial community, reduction of AMR risk, and efficiency against multidrug-resistant bacteria and biofilms when compared to antibiotics [124]. The benefits of endolysin therapy have attracted the attention of researchers and pharmaceutical companies to its commercial potential and several commercial products based on endolysin have now been developed. The first human endolysin product developed by Micreos, Staphefekt SA. 100, specifically for the treatment of chronic *S. aureus* associated skin diseases, has been marketed. All three clinical patients had a positive therapeutic effect and did not develop resistance [125]. Artilysin has developed an Artilysin^®^ product line(Lysando AG, Regensburg, Germany) that is effective against resistant *P. aeruginosa* and *A. baumannii* in various forms including spray, nebulizer, solution, lyophilization, gel, and coating [126]. Rephasin^®^ SAL200 (Intron Biotechnology, Seongnam, Korea) is now in phase II of human clinical trials [105,127]. ContraFect has developed a novel direct lysing agent called Amurin peptide, which is effective against numerous Gram-negative pathogens. The other is a lysin-based direct lytic agent, containing Exebacase CF301, which is effective against *S. aureus*, including MARS, and is the first phage lytic enzyme to enter human clinical trials in the United States [128,129]. Criteria used for the preclinical analysis of small molecule antibiotics may be more readily translated into the preclinical assessment of phage lytic enzymes than phages, so clinical evaluation of phage lytic enzymes is progressing significantly faster [130].

## 7. Advantages and Disadvantages of Phage Therapy

Compared to antibiotics, phages are characterized by host specificity, which means only lysing the host bacterial cell wall without destroying the microbiota [62]. There is a process of adaptation versus counter-adaptation in the coevolution of phages and bacterial hosts and the risk of developing resistance is low [40]. For example, coevolutionary phage training can delay the evolution of phage resistance. Researchers conducted coevolution experiments using *E. coli* and untrained or trained phages to assess the potential of phage-training treatments and found that trained phages were able to inhibit host bacteria for a longer period of time [131]. The coevolution of phages with host bacteria has also driven bacteria to evolve a variety of highly specific phage defense mechanisms. For example, mutations in phage receptors, the R-M system, the DISARM system, the superinfection exclusions (SIEs) system, the abortive infection (Abi) system, and the adaptive immune system CRISPR-Cas all make phages resistant [132,133]. Hussain studied the evolutionary trajectory of resistance in wild-type phages, which showed that the rapid evolution of mobile phage defense elements (PDEs) drove bacterial resistance to phages [134]. Studies have shown an evolutionary trade-off between phage and antibiotic resistance, with bacteria sometimes showing increased susceptibility to antibiotics when phage resistance evolves. Barber studies have demonstrated that efflux pumps play a dual role in antibiotic resistance and phage sensitivity, and when phage resistance leads to the loss of bacterial capsules, they will subsequently become sensitive to antibiotics [135]. However, when Burmeister studied *E. coli* phages, it was found that bacterial interaction with phages may depend on efflux pump protein TolC and structural barrier molecule lipopolysaccharide (LPS), and when these two mutants were constructed, some phage resistance mutations conferred an increase in antibiotic resistance [136]. Therefore, there are not only synergistic effects but also antagonistic effects between phages and antibiotics, and their intrinsic mechanisms of action remain to be further studied. In addition, phages replicate only in the target bacteria at the site of infection and treatment causes fewer adverse effects and is safer. Oral phage preparations are generally harmless and researchers have found the presence of adverse effects associated with phage therapy when assessing animal and clinical phage therapy safety and toxicity, but with a small probability of events [137]. Finally, phages are widespread in the environment and provide an inexhaustible resource. It only takes days to weeks to produce a new natural phage preparation, and if a phage develops resistance, phages that use other new receptors can also be quickly found. Screening for a new natural phage preparation takes a few days, and phages using other new receptors can also be quickly found if the phage develops resistance.

Despite the favorable results of various studies on phage applications, phage therapy still has certain shortcomings and unknowns. First, there are still some potential risks associated with the application of phage therapy, which are largely observational with existing phage therapies or performed in small non-randomized trials, where side effects may be underestimated. Second, the route of administration, frequency of administration, dose, phage resistance, pharmacokinetic and pharmacodynamic characteristics of phages, and the mechanism of phage entry into eukaryotic cells and the immune system need to continue to be studied in depth [40,138]. Third, legal regulation is a significant obstacle to the implementation of phage therapy and regulatory authorities classify phages as biological substances, which differs from the approval and production of antibiotics, making it difficult to use phage therapy. European legislators have been advocating for a regulatory framework specifically targeting individualized phage preparations but they have been strongly resisted [139]. Fourth, considering phages are natural entities, they entrap pharmaceutical companies in intellectual property issues [121]. Fifth, animal prophylactic phage products do not remove phages immediately after use and may lead to phage mutation and the cultivation of phage mutants. This problem also needs to be solved by using the regular rotation of phages and continuous detection, such as antibiotics [35]. Sixth, phages can transfer bacterial resistance genes and even contain toxic genes, implying that, as much as possible, the selection of lytic phages ensures that therapeutic phage products must be deeply purified and must remove endotoxins during processing [140]. Seventh, when the scope of phage application expands, including antibiotic substitutes, carrier delivery drugs, vaccines, and phage display technology, the demand for large-scale production of phage increases. The Phage on Tap (PoT) protocol has been studied for the rapid formulation of high titer phage formulations and a systematic procedure has also been developed for the isolation, up-culture, concentration, and purification of phages for pharmaceutical use [141]. The procedure can combine modified classical techniques, modern membrane filtration processes, and no organic solvents in 16 to 21 days, producing an average of 23 mL of 10^11^ PFU/mL phage [142]. Despite the enormous efforts of researchers for phage technology, there remain challenges for the production and expansion of wild-type phages for biological control. Finally, doctors and the public at large are unaware of the use of phages to treat diseases and the public believes that viruses are exclusively harmful to the human body, not realizing that they may also be beneficial [143].

## 8. Conclusions and Prospects

**Figure 3:**
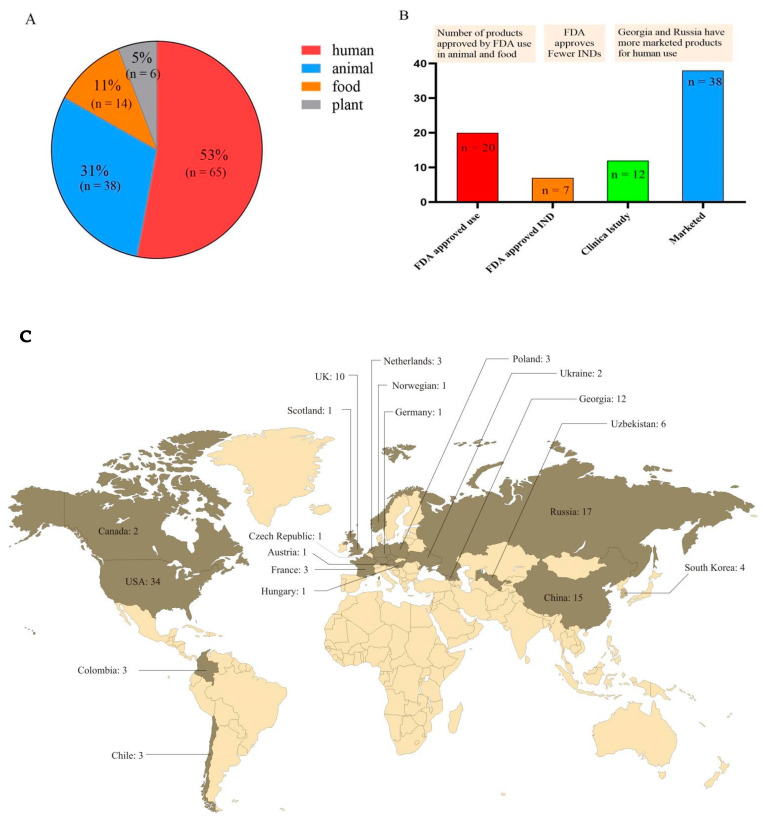
Total phage products are distributed and approved. (**A**) The proportion of phage products in plants, animals, food, and humans. (**B**) The number of phage products that have been approved by the FDA and are in clinical research and marketed for human use. (**C**) Worldwide distribution of the number of phage products.

Antibiotic resistance poses a threat to global health. Russia approved the addition of phages to the official pharmacopoeia in 2016. The European Pharmacopoeia included “phage therapeutic active ingredients and pharmaceutical products for human and veterinary use” in 2021. To ensure its safety and effectiveness, pharmaceutical authorities such as the FDA and EMA require that any modern phage therapy product meet GMP standards, which poses challenges [144]. According to the statistics, Figure 3C reveals that 20 countries began to develop phages and had phage products approved for use. According to the analysis of 123 products in 20 nations, Figure 3A reveals that 53% of the products were used for human health, and Russia, Georgia, and the United States have rich experience in phage therapy for human diseases. Figure 3B reveals that the FDA has approved twenty phage products for animals and food, but only seven investigational new drugs (INDs) for humans. Except for Russia and Georgia, which have focused on phage therapy for human diseases and have sold many phage products, phage product research and development in other nations remains to be further developed. Overall, phage products in the United States are rapidly developing and the FDA has also approved several products. In the future, as a novel alternative therapy under the “one health” approach, phage research and development will continue to focus on making products that are environmentally friendly, safe, and successful in combating AMR.

## Figures and Tables

**Table 1:** Commercial phage products for plant health.

Target Bacteria	Company	Products	Regulatory Approval	Certifications	Application	Ref.
*Soft rot Enterobacteriacea*	APS Biocontrol Ltd. (SCO)	Biolyse^®^ BP	Approved	UK, European	Food processing assistants in the potato packaging industry	[34]
*Clavibacter michiganensis*	OmniLytics Inc. (USA)	AgriPhage CMM™	EPA approved	USA, Canada	Tomato bacterial canker	[35,36]
*Xanthomonas citri*		AgriPhage™ Citrus Canker			Citrus canker	[27]
*Erwinia amylovora*		AgriPhage™ Fire Blight			Fire blight for apples and pears	
	Erwiphage PLUS (HU)	Erwiphage	Undefined	Hungary	Fire blight caused by plants in the rose family	[37]
Variety of bacteria	Fixed-Phage (UK)	agriPHIX™		UK	Effective improvement of storage for a range of crops	[38]

**Table 2:** Commercial phage products for animal health.

Target Bacteria	Company	Products	Regulatory Approval	Certifications	Application	Ref.
*E. coli*	Intralytix (USA)	Ecolicide^®^	FDA	USA	For *E. coli O157:H7* contamination in Pet Food	[48]
		Ecolicide PX™			For *E. coli O157:H7* contamination on animal fur	
	Arm and Hammer Animal & Food Production (USA)	Finalyse^®^	USDA, FSIS	USA	A preharvest antimicrobial hide wash used to reduce *E. coli O157:H7*	[34]
	Proteon Pharmaceuticals (POL)	BAFACOL™	EFSA	Poland	Feed additive to prevent pathogenic *E. coli* in poultry.	[49]
	Phagelab (CHI)	Swine product	Undefined	Chile	Liquid Food Additive Eliminates *E. coli* in Swine	[50]
*Salmonella*	Intralytix (USA)	SalmoLyse^®^	FDA	USA	*Salmonella* Contamination in Pet Food	[51,52]
		PLSV-1™			*Salmonella* Contamination in Poultry	[44,53]
	Proteon Pharmaceuticals (POL)	BAFASAL+G^®^	EFSA	Poland	Feed additive to treat the digestive tract of poultry	[51,54]
	UniFAHS	SalmoGuard	FDA	Southeast Asian countries	Poultry feed additives	[52,55]
	OmniLytics Inc. (USA)	BacWash™	USDA	USA	For Hides of livestock surface disinfection	[56]
	SciPhage (CO)	SalmoFree^®^	Undefined	Colombia	Feed additive for control of *Salmonella* infection in poultry	[57]
	PhagePharm (CHN)	NuoAnSha	Approved	China	Improve the breeding environment	[58]
	Phagelab (CHI)	Poultry product	Undefined	Chile	Liquid food additive to eliminate *Salmonella* in broilers.	[50]
*L. monocytogenes*	Intralytix (USA)	ListPhage™	FDA	USA	*L. monocytogenes* in pet food	[48]
*C. perfringens*		INT-401™	FDA, FSIS		Against Poultry *C.* *perfringens*	[59]
	PhagePharm (CHN)	NuoAnSuoQing	Approved	China	Necrotizing enteritis, diarrhea, intestinal bleeding caused by *C. perfringens*	[58]
*staphylococcus*	Delmont Laboratories (USA)	Staphage Lysate(SPL)^®^	FDA	USA	*Staphylococcal* skin infections in dogs	[47]
*Yersinia ruckeri*	ACD Pharma (NOR)	CUSTUS^®^_YRS_	FOT	Norwegian	Various bacteria in aquaculture farms	[60]
*R. anatipestifer*	PhagePharm (CHN)	JiangYanQing	Approved	China	Decontamination of R.anatipestifer in aquaculture environments	[58]
*Weisella ceti*	SciPhage (CO)	Weissella Ceti Phages	Undefined	Colombia	control *weissellosis* in trout	[61]
Variety of bacteria	CJ CheilJedang Research Institute of Biotechnology (KOR)	Biotector^®^S	Undefined	South Korea	Feed additive for poultry and pigs against *Salmonella, C.perfringens, E. coli.*	[35]
	Phagelab (CHI)	cattle product		Chile	Food additive prevent infectious diarrhea caused by *E. coli* and *Salmonella*.	[50]
	Proteon Pharmaceuticals (POL)	BAFADOR^®^	EFSA	Poland	Fish feed additive against *Aeromonas* and *Pseudomonas*	[62]
	PhagePharm (CHN)	NuoAnQing	Approved	China	Improve the breeding environment	[58]
		YaLiNing				
	Varmsphage (CHN)	ChangShi			Infections caused by *E. coli* and *Salmonella*	[63]
	Cytophage (CAN)	Poultry Feed Additives	Undefined	Canada	Prevents the common bacterial infections in chickens	[64]
		swine bacteriophage			Against the common bacterial infections in swine	
	Fixed-Phage (UK)	aquaPHIX™	Approved	UK	Added to the feed as a solvent	[37,49]
		farmPHIX™			Feed additives	
		petPHIX™			Topical application of gels and creams	
	Pathway Intermediates (KOR)	ProBe-Bac	FDA	South Korea	ProBe-Bac SE for pigs; ProBe-Bac PE for poulty	[65]
	Phagelux (CHN)	LUNIN	Approved	China	for poultry diseases	[66]
		LUZON			for swine disease	
		LUMON			for cattle disease	
	MicroMir (RUS)	Vetagin^®^	Approved	Russia	Prevention of bacterial endometritis, abscess and myositis in dairy cows	[67]
		Bronchophage			Control of common bacteria associated with lower respiratory tract disease	
		Phagovet			Prevention of bacterial diseases in broilers	

**Table 3:** Commercial phage products fighting foodborne pathogens in food.

Target Bacteria	Company	Phage Products	Regulatory Approval	Certifications	Application	Ref.
*E. coli*	Intralytix (USA)	EcoShield PX™	FDA	Canada; Israel; USA	Eliminate *E. coli O157:H7* contamination prior to grinding or packaging	[71,75]
	Micreos (NED)	PhageGuard E™		USA	*E. coli O157* on beef carcasses, primals, subs and trimmings.	[76]
	FINK TEC GmbH (GER)	Secure Shield E1			Used in beef products, turkey and other foods	[48]
*Salmonella*	Intralytix (USA)	SalmoFresh™		USA, Canada, Israel	Food additives for poultry, fish, shellfish, fruits and vegetables.	[77]
	Micreos (NED)	PhageGuard S™		Canada; Israel; Halal; OMRI; SKAL	In spray or dipping form for poultry, meat.	[78]
	Phagelux (CHN)	SalmoPro^®^		Canada, China	As an antibacterial processing aid in food.	[56]
	Arm and Hammer Animal & Food Production (USA)	Finalyse™SAL	Undefined	USA	For *Salmonella* in poultry products.	[79]
*L.* *monocytogenes*	Intralytix (USA)	ListShield™	FDA	USA	Food additives for poultry, fish, shellfish, fruits and vegetables.	[80]
		Listex™				[81]
	Micreos (NED)	PhageGuard Listex™		Swiss; Israel; Halal; Canada; Kosher OMRI; SKAL	In spray or dipping form for poultry, meat.	[82,83]
*Campylobacter*	Intralytix (USA)	Compyshield™		USA	Food additives for raw red meat	[84]
*Shigella*	Intralytix (USA)	ShigaShield™			Removal of *Shigella* from meat and vegetables	[53,85]
Variety of bacteria	Brimrose Technology Corporation (USA)	EnkoPhagum	Approved		*Salmonella, Shigella, E. coli, Staphylococcus* in meat products.	[53]
	Fixed-Phage (UK)	safePHIX™	Undefined	UK	Against bacteria in the food cold chain	[38]

**Table 4:** Phage products for human health.

Mono Component						
Target Bacteria	Company	Product	Regulatory Approval	Route of Administration	Application	Ref.
*E. coli*	Intralytix (USA)	EcoActive™	FDA approved IND, Phase 1/2a	oral	Targeting adherent-invasive *E. coli*	[98]
	Pherecydes Pharma (FRA)	PhagUTI	Phase I/II	Undefined	Treating *E. coli* Urinary Tract Infections	[99]
	Phico Therapeutics (UK)	SASPject PT5	Uundefined	Intravenous injection	Fights diseases caused by *E. coli*	[100]
*P. aeruginosa*	Microgen (RUS)	Bacteriophage *P. aeruginosa*	Russian Federation national standard certification	Oral intrarectal, or Intracavitary injection	Treatment and prevention of diseases caused by *P. aeruginosa*	[101]
	Armata (USA)	AP-PA02; AP-PA03	FDA approved IND, Phase 1b/2	Inhalation	Treatment of respiratory tract infections caused by *P. aeruginosa*, especially in patients with CF	[95]
	BiomX (USA)	BX004	Preclinical	Oral		[102]
	Phagelux (CHN)	PGX0100	FDA approved IND, preclinical	Transdermal	Spray and gel for burn care	[103]
	Phico Therapeutics (UK)	SASPject PT3	undefined	Undefined	Against *P. aeruginosa* infection	[104]
	Pherecydes Pharma (FRA)	Pneumo Phage	Phase I/II clinical trials are expected to start in 2023	Inhalation	Treatment of acute *P. aeruginosa* respiratory tract infection	[99]
*S. aureus*	Microgen (RUS)	Staphylococcal bacteriophage	Russian Federation national standard certification	Inhalation	Treatment of Suppurative Inflammation and Intestinal Disorders Caused by *Staphylococci*	[101]
	Armata (USA)	AP-SA01; AP-SA02	FDA approved IND, Phase 1b/2	Intravenous injection	Treatment of resistant and refractory *Staphylococcus* aureus bacteremia and diabetic foot ulcers	[93,94]
	BiomX (USA)	BX005	Preclinical stage	Transdermal	Atopic dermatitis caused by *S. aureus*	[102]
	Phagelux (CHN)	PL-01-SZ	China NMPA IND submission expected in 2022		*S.aureus* lyase, a hydrogel formulation for the treatment of eczema	[103]
		PL-06-FC			*P.acnes* and *S.aureus* lyase, hydrogel for acne treatment	
	iNtODEWorld (KOR)	N-Rephasin^®^ SAL200	Phase II	Intravenous injection	Effective against MRSA	[105,106]
	Pherecydes Pharma (FRA)	Phage Cocktail	Phase I/II	Undefined	Fights bone and joint infections (IOA) and diabetic foot ulcers (UPD) caused by *S.aureus.*	[99]
	Phico Therapeutics (UK)	SASPject PT1.2	Phase I		Engineered phages deliver genes for antimicrobial proteins (SASPs) that rapidly kill *S. aureus*	[107]
*Staphylococcal*	Eliava Bio Preparation (GEO)	Staphylococcal Bacteriophage	Georgian Approval	Oral or intrarectal	Prevention and treatment of postoperative wound infections, *Staphylococcal* infections	[108]
*K.pneumoniae*	BiomX (USA)	BX003	Phase I	Oral	Targeting *K. pneumoniae* bacterial strains present in the gut of IBD and PSC patients	[102]
*Shigella*	Intralytix (USA)	ShigActive™	FDA approved IND,2021	Oral	Prevention of human diseases caused by *Shigella* infection	[109]
*Enterococcus*		VRELysin™	Undefined	Undefined	Colonization with antibiotic-resistant *Enterococci* and associated bacteremia	[84]
*C difficile*	AmpliPhi (UK)	AmpliPhage-004	Pre-phase 1	Undefined	Against *C. difficile* (including highly virulent RT027)	[104]
*Streptococcal*	Microgen(RUS)	Streptococcal bacteriophage	Russian Federation national standard certification	Oral, topical and intrarectal	Treatment diseases caused by *Streptococcus*	[101]
*Gardnerella spp*	BioNTech R&D(AUT)	PM-477	Preclinical	Undefined	Recurrent bacterial vaginosis, synthetic lysosomes	[110]
*Fusobacterium* *nucleatum*	BiomX (USA)	engineered phage	Preclinical	Intravenous injection	Targeting *Fusobacterium nucleatum* bacteria present in the tumor micro environment.	[111]
**Combining targets against variety of bacteria**						
Dosage Form	Company	Product	Regulatory approval	Route of administration	Application	Ref
Phage spray	Biochimpharm (GEO)	Phagyo^®^spray	Georgian Approved	Topical	Treatment and prophylaxis of bacterial purulent–inflammatory infections (multiple microorganims)	[112]
Phage tablet		Septaphage^®^table		Oral		
Phage cocktail		Septaphage^®^				
		Phagyo^®^				
		PhageStaph				
Phage capsule		Travelphag™			For bacterial infections, indigestion	
Phage cocktail	Microgen (RUS)	*Salmonella* groups A,B,C,D, bacteriophage	Russian Federation national standard certification	Oral, intrarectal	Treatment and Prevention of Diseases Caused by *Salmonella*	[101]
		*E.coli*-Proteus bacteriophage		Oral, topical and intrarectal	Treatment and prevention of purulent inflammatory and enteric diseases, dysbacteriosis caused by bacteria Proteus and enterotoxigenic *E.coli*	
		Klebsiella purified polyvalent bacteriophage			Specific lysis of *K. pneumoniae*, *K. odorifera*, *K. rhinosclerosis.*	
		Intesti-bacteriophage			Treatment and prevention of bacillary dysentery	
		Sextaphage ^®^ Polyvalent Pyobacteriophage			Treatment and prevention of purulent inflammation and intestinal diseases	
		Complex Pyobacteriophage			Specific lysis of *Staphylococcus, Streptococcus, Enterococcus, Proteus, K.pneumoniae, P.aeruginosa* and *E. coli.*	
		Dysentery polyvalent bacteriophage		Oral and intrarectal	Specific lysis of the bacillary dysentery pathogen	
Phage cocktail	Eliava Bio Preparation (GEO)	Pyo-Phage	Georgian Approved	Oral, intrarectal, or intracavitary injection	Treatment and prevention of bacterial purulent inflammation and intestinal infections.	[113]
		Fersisi-Phage				
		Intesti-Phage		Oral or intrarectal		
		SES-phage		Rectal, or intracavitary injection		
		ENKO-Phage		Oral		
Phage spray	Aziya Immunopreparat (UZ)	Bacteriophage Staphylococcus spray MediPhag	Marketed	Topical(spray)	A mix of sterile lysate phages against *S. aureus*	[114]
Phage cocktail		Bacteriophage Staphylococcus liquid MediPhag		Oral		
		Bacteriophage Salmonella polyvalent MediPhag			Treatment and prevention of multiple serotypes of *Salmonella*	
		Bacteriophage Dysenteric Polyvalent MediPhag			A mix of sterile lysate phages against *Shigella*	
		GastroFag polyvalent MediPhag			Fight enteric diseases such as *Salmonella, Proteus, S.aureus, P.aeruginosa, E.coli*	
Phage capsule		Bacteriophage dysenteric polyvalent “MediPhag”			A white gelatin capsule containing lyophilizied dried bacteriophage capsules against *Shigella*	
phage tablet	MB Pharma (CZ)	LYZODOL^®^	Marketed	Oral	Against *S.aureus, K.pneumoniae*, *Lelliottia amnigena*, *Propionibacterium acnes* causing respiratory infections.	[115]
Phage gel	MicroMir (RUS)	Phagodent	Marketed	Topical	Contains 72 phage complexes to normalize oral microflora	[76]
		Phagoderm			Skin gel containing 64 phages to prevent bacterial infection of the skin.	
		Phagogyn			Gel containing 74 phages that prevent bacterial diseases of the reproductive system.	
		Otophagus			Gel containing 69 phages that prevent bacterial and suppurative inflammation of the ear, nose and throat	
Phage cocktail	Phagex (UKR)	Pyofag^®^	Marketed	Oral and topical	Treatment of pathogenic factors in purulent inflammation and intestinal diseases caused by *Streptococcus pyogenes, S.aureus, E.coli, P.aeruginosa, Proteus vulgaris, Proteus mirabilis*	[116]
		Intestifag^®^ polyvalent bacteriophage			Fights intestinal diseases caused by *Shigella, Salmonella, E. coli, P. aeruginosa, Enterococcus faecalis, S. aureus*	
	Phico Therapeutics (UK)	SASPject PT4	Undefined	Intravenous injection	Treatment and prevention of diseases caused by K. pneumoniae and *E.coli*	[100]
	Phagelux (CHN)	BACTELIDE™	FDA approved IND, preclinical	Transdermal	Patches and sprays for pressure ulcers	[103]
	Fixed-Phage (UK)	mediPHIX™	Undefined	Undefined	Effective against a variety of bacteria	[38]
	Adaptive Phage Therapeutics (USA)	PhageBank	FDA approved IND, Phase 1/2	Intravenous injection	Treat Diabetic Foot Osteomyelitis, Prosthetic Joint Infection, Chronic Recurrent UTI, Ophthalmic Infection, Cystic Fibrosis-related Lung Infection	[117]
	Locus Biosciences (KOR)	crPhage™	Phase 1b	Injection	Combined with CRISPR-Cas3 to enhance bactericidal efficacy against various bacterial diseases such as IBD and UTI	[118]
	Ellis Day Skin Science (USA)	Balancing Phage Serum	Marketed	Transdermal	Eliminate bacteria associated with blemishes and acne to balance the skin microbiome	[119]
		Hydrating Phage Serum				
	PHYLA (USA)	Phortify Probiotic Serum	Marketed		A probiotic serum that targets and neutralizes acne-causing bacteria	[120]
	SciPhage (CO)	AcneFree	Undefined		Fights acne-targeting bacteria	[61]

## Data Availability

Not applicable.
